# Challenges of minimally- invasive orthopaedic surgery practice in Nigeria - a national survey of residents' perceptive

**DOI:** 10.4314/ahs.v24i3.44

**Published:** 2024-09

**Authors:** Kelechi Imediegwu, Favour N Emmanuel, Olikagu Felix, Kenechukwu J Okonkwo, Chinonso J Dimson, Ugwu Oge, Bobby D Edeani, Winifred A Acho

**Affiliations:** 1 National Orthopaedic Hospital, Enugu, Nigeria, Orthopaedic surgery; 2 UNTH, College of Medicine

**Keywords:** Minimally- invasive orthopaedic surgery, residents' perceptive, Nigeria

## Abstract

**Background:**

Minimally invasive orthopaedic surgery (MIOS) practice globally has been on a gradual increase, however the current state in Nigeria is not same.

**Objectives:**

To determine the challenges of minimally invasive orthopaedic surgery (MIOS) practice in Nigeria and proffer realistic suggestions to improve the current state in Nigeria.

**Methodology:**

A descriptive cross-sectional online survey conducted among senior orthopaedic surgery residents across all specialist hospitals in Nigeria. Data was analysed with the SPSS software version 20. Significance was set at p<0.05. Mean and S.D scores were calculated for responses with Likert scales of 1-5 (5- strongly agree).

**Results:**

48 residents completed and submitted the questionnaire, response rate of about 70.6%. The results showed that the most significant factors affecting minimally invasive orthopaedic surgery practice in Nigeria were lack of funds (72.9% of respondents), unavailability of equipment and implants (60.4%), limited number of trainers and fellows skilled in minimal access surgery (54.2%). The narrowest Standard deviation reflecting closest precisions in perspectives of the challenges was a S.D of 0.794, Mean 4.02, which stated that there are very few courses on training of MIOS procedures.

**Conclusion:**

Funds, training, equipment availability were the major challenges of minimally invasive orthopaedic surgery practice in Nigeria.

## Introduction

The desire of every surgeon in patient's management is to employ the surgical technique with minimum local and systemic damage and maximum benefit^1^. This has been made achievable with the emergence of minimally invasive surgery.

Minimally invasive surgery (MIS) is aimed at using the least amount of stimulus to achieve therapeutic and diagnostic surgical outcomes with minimized metabolic, physiologic and cardiorespiratory effects.^2^ Minimally invasive surgery (MIS) has been developed since the late 1980s and it has been regarded as one of the most important achievements in modern medicine.^3^ Ever since the emergence of minimal invasive surgery into modern practice, it has increasingly been adopted in many sub-specialties in surgery and orthopaedic surgery is not left out.

Minimally invasive orthopaedic surgery (MIOS) practice globally has been on a gradual increase. However the current state in Nigeria is not the same. Even though a few MIOS cases such as Mini-incision for bankart repair, mini-open rotator cuff repair, minimally invasive approaches to arthroplasty are gradually been done in some Nigerian hospitals especially in the private sector, some other minimally invasive orthopaedic surgery procedures like arthroscopic ankle arthrodesis, mini-incision shoulder arthroplasty, custom total/patient specific total knee replacement, computer-guided and computer navigation total hip arthroplasty are rarely reported, and these are amazing trends to look forward to. The use of MIS in fracture care is not left out, as minimally invasive treatments of greater tuberosity fracture care is now operational and the use of arthroscopes for reduction of tibial plateau fractures is also now routinely done in some developed countries. Modern surgical practice achieves same or better surgical objective with technology-based techniques ensuring stimulation of minimal body responses. This is akin to what happens in other disciplines outside medicine where present-day technology has impacted positively on practice with resultant improvement in outcome.^4^ Minimally invasive surgery offers great benefits to patients over conventional open surgery, the major benefits include reduced surgical trauma, reduced wound complications, shorter hospital stay, accelerated recovery, less postoperative pain, fewer operative and post-operative complications, less scarring, less stress on the immune system, smaller incision, reduced operating time and reduced costs.^5^ Surgical practice has evolved noticeably owing to the emergence of minimally invasive surgery (MIS) from conventional open surgery.^6^ However, MIS is technically more demanding because the surgical intervention is executed remotely via two-dimensional imaging of the operative field, with loss of tactile feedback, restricted maneuverability and less efficient control of major bleeding.^3^

In this unique study, challenges to specific MIS in orthopaedics in Nigeria such as arthroplasty, endoscopic spine surgery and arthroscopy would be noted in depth. This study aims to explore and establish the limitations to MIS in orthopaedics ranging from personnel to skill acquisition and government roles in Nigeria, which has not been clearly documented in existing literatures and proffer realistic solutions/recommendations.

## Materials and methods

### Study Area

This study was conducted amongst all orthopaedic surgery residents in the three (3) National Orthopaedic Hospitals in Nigeria. These are the National Orthopaedic Hospital, Dala, Kano state; National Orthopaedic Hospital, Enugu, Enugu state and the National Orthopaedic Hospital, Igbobi, Lagos state, Nigeria.

### Study Type

This is a descriptive cross-sectional online survey conducted among senior orthopaedic surgery residents across all Nigerian teaching hospitals and National Orthopaedic Hospitals in Nigeria in order to determine their challenges with minimally invasive orthopaedic surgery.

### Study Population

The study population included all senior specialist orthopaedic resident doctors across all teaching hospitals and national orthopaedic hospitals in Nigeria. Survey responses were collected anonymously.

### Sampling Method

The sampling procedure was convenience sampling method as senior orthopaedic surgery residents from all teaching hospitals and the three national orthopaedic hospitals in Nigeria were allowed to participate in the study. Participating doctors however voluntarily consented to participate and the questionnaires were sent to the various resident's social media platforms.

### Study Design

A predesigned online-based questionnaire was developed by the principal investigator. The content accuracy and internal validity of the survey items were finalized with multidisciplinary input from the study investigators. A pilot study was carried out among 20 orthopaedic surgery residents in selected hospitals across the country. Data was collected using a semi-structured online-based questionnaire created on Google forms.

The questionnaires had two (2) sections. Section one (1) assessed socio-demographic characteristics of the respondents like age, sex, marital status, religion and level of training.

Section two (2) assessed challenges of minimally invasive orthopaedic surgery. It included questions on availability of equipment: low stocking and scarce equipment spare parts, lack of funds for the procurement of equipment/instruments, lack of functionally enabling environment which includes steady light, available water and technical facilities. It also included questions on government policies, cost of training staff to handle sophisticated equipment, inability of patients to afford procedures, late presentation of patients that make these procedures largely inapplicable, limited number of trainers or fellows skilled in minimally invasive procedures, low interest of surgeons to train in minimally invasive procedures, having few courses for skill acquisition on minimally invasive procedures, lack of sponsorship to update courses on minimally invasive surgery, lack of sponsorship to attend conferences on minimally invasive surgery and fellowship training opportunities on minimally invasive procedures are limited and expensive.

**Limiting Bias-** Measures to limit bias were ensured by randomization of options in the questionnaire (this limited answer order bias); increased call-backs to improve response rate and limit response bias. Two independent analysts were recruited to reduce systematic errors. A Pilot Study was also done with 20 residents.

### Response rate

The estimated number of orthopaedic surgery senior residents in Nigeria as at the time of the study on all online platforms was 68. A total of 48 responses (response rate - 70.6%) were gotten from the online Google form questionnaire filled and submitted.

### Data Analysis

Data was analysed using the IBM Statistical Package for Social Sciences (SPSS) and results were presented in graphs, pie charts and tables. Descriptive and inferential analysis was done. Significance set at p<0.05. Mean and S.D scores were calculated using Likert scales of 1-5.

### Ethical Approval

Ethical approval was obtained from the Health, Research and Ethics Committee of the University of Nigeria, UNTH, Enugu, Nigeria with the REF NO- UNTH/HREC/2022/06/464.

## Result

### Socio demographic characteristics of respondents

At the end of the study, forty eight (48) orthopaedic surgery residents out of about sixty eight (68) in all Nigerian teaching hospitals compiled from a list of residents from the last four (4) national postgraduate surgery exams filled and submitted the questionnaire. Amongst the respondents, a larger number of respondents were in the age group of 31-35 years and most were found to be males as shown in Fig 1. Most of the respondents are married. Majority occupied the senior registrar level 2 cadre (55.1%)

**Figure:**
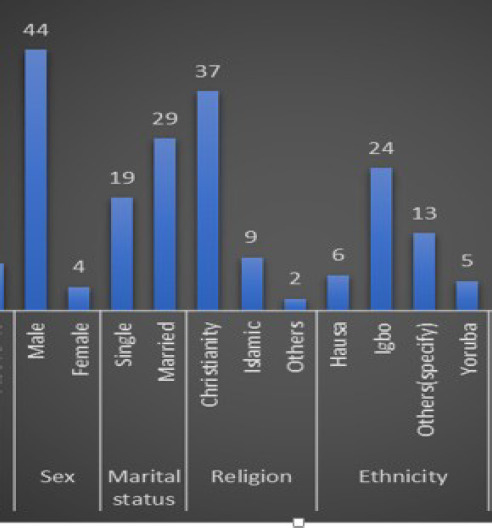
Socio-demographics

### Challenges of minimally invasive orthopaedic surgery in Nigeria

Using the Likert scale of 1-5, for which 1 is disagree and 5 is strongly agree, the analysis of our study shows that the major significant factors affecting minimally invasive orthopaedic surgery practice in Nigeria were lack of funds (72.9% of respondents, Average Mean value – 4.13), unavailability of equipment and implants (60.4%, Average Mean value - 4.59), lack of good functionally enabling environment (54.2%; Average Mean value - 4.46), limited number of trainers and fellows skilled in minimal access surgery (54.2%; Average Mean value - 4.32), poor financial strength of the patients (50%; Average Mean value -4.15), limited and expensive training opportunities on minimal access orthopaedic surgery (47.9%; Average Mean value - 4.21).

**Figure:**
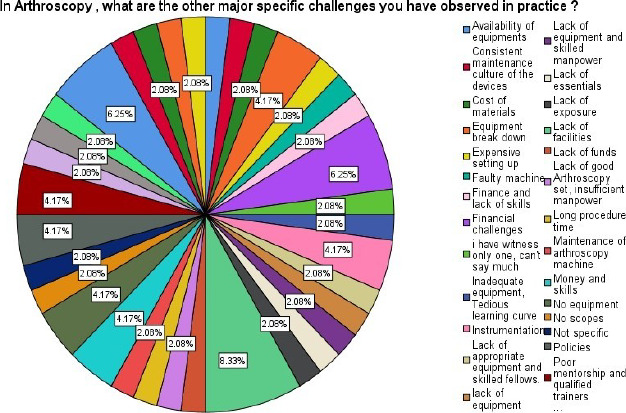
Specific challenges of arthroscopy

**Figure:**
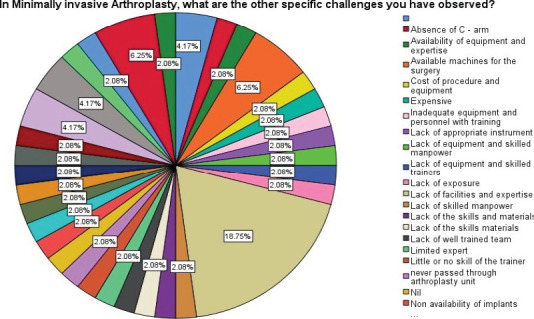
Challenges of Arthroplasty

**Figure:**
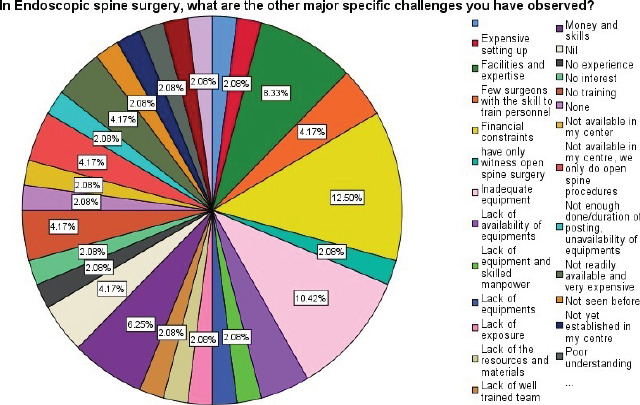
Challenges of spine surgery

The interpretation of the figure 4 above is as follows:

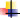
Strongly Agree=Mean of 4.21–5.00
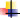
Agree=Mean of 3.41–4.20
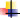
Not sure=Mean of 2.61–3.40
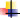
Disagree=Mean of 1.81–2.60
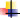
Strongly Disagree=Mean of 1.00–1.80

## Discussion

This study showed that the most common challenges that affect minimally invasive orthopaedic surgeries were non-availability of equipment, lack of funds and functionally enabling environment, limited number of trainers and fellows skilled in minimal access surgery, limited and expensive training opportunities on minimal access surgery. Since the 1980s, technological advancement and innovation have seen surgical techniques in MIS rapidly grow as it is viewed as more desirable^7^, compared to routine open approaches. This prompts the need to understand the challenges that affect MIS in orthopaedics in Nigeria.

Topmost amongst the agreed challenges was unavailability of equipment and implants which is common in most regions of the country (Nigeria) with a mean precision of 4.59 +_ 0.617 obtained from the respondents. (Key-Strongly Agree = Mean of 4.21–5.00). In that regards, very few centres in Nigeria can produce sterile orthopaedic implants for this procedures which is a major challenge. This is because manual method of cleaning of surgical instrument is still employed in middle and low income countries as outlined in a study done in Brazil by Dayane de Melo Costa et.al.^8^

Another major highlighted challenge from our study was the lack of available sponsorship for trainees who are willing and limited number of skilled trainers which have posed to be a hurdle to the acquisition of MIS skills in Nigeria. This is in tandem to the findings of Ijah et.al among minimal invasive surgeons where it was noted that only a hand full of surgeons in a scientific conference from 14 African countries in 2019 made use of laparoscopy in their practice.^4^

These challenges seem to look like an “African” problem as the factors affecting MIOS in Nigeria are similar to those identified in other African countries. This is found to differ in developed countries where the use of minimal invasive surgeries are the trend now. In a study done by Treuting et.al among patients who had arthroscopic surgeries, the global most important innovation in orthopaedic surgery are in joint replacement arthroplasty and arthroscopy^9^, and efforts should be made to improve provision of minimally invasive procedures in this cases to the citizens to limit medical tourism to the barest minimum in Nigeria especially as our study showed that 72.9% of the respondents significantly agreed that cost, funds are quite deficient and needs to be improved on to be able to allow arthroplasty and arthroscopy practice in Nigeria to meet up with this global trends.

Skill acquisition is notably low as agreed by 27.1% of the respondents and this could possibly be due to the low number of available cases which would ultimately affect the competence of the surgeons for those quality of cases such as in minimally invasive arthroplasty cases. This is supported and of same view by a study done by Seyler et.al on Arthroscopic–assisted minimally invasive total knee arthroplasty which revealed that the use of an arthroscope would allow intraoperative identification of potentially adverse findings including bone and cement fragments.^10^

A possible way forward is partnering with some recognized hospitals in Europe and organizations to organize and engage local surgeons in hands-on-programs. These challenges when addressed will help improve the use of MIOS in Nigeria and evolve orthopaedic surgical practice.

## Conclusion/recommendations

Minimal invasive orthopaedic surgery has been adopted in so many developed countries but has been shown to relatively deficient in Nigeria. Unavailability of equipment, instruments, lack of tutors, unfavourable government policies and lack of sponsorship has been identified as the barrier towards its achievement in Nigeria.

Based on the findings of our study, we recommend that Government and responsible agencies should allocate more funds to the development of training centres for orthopaedic specialists and residents to learn more about minimal invasive orthopaedic surgery.

Furthermore, fellowship programs and minimally invasive courses especially navigation, computer- assisted courses should be sponsored at regular intervals and the Government should make available the necessary equipment needed for this procedure to the hospitals.

In addition, these suggestions can be implemented by partnerships with established organizations like the British Orthopaedic Association (BOA), American Academy of Orthopaedic surgeons (AAOS) and the AO Trauma Group with a comprehensive nationwide campaign and training of surgeons with senior registrars. Also, more collaborations with implant companies would also help for organizing skill courses and fellowship attachments which could hold yearly or biannually.

Older skilled consultants in minimally invasive procedures should also be available to teach the resident doctors at every opportunity to help grow their interest in this new technique. In conclusion, patients should be educated to present early in the hospitals so that these procedures can be beneficial to them.

## Figures and Tables

**Figure 5:**
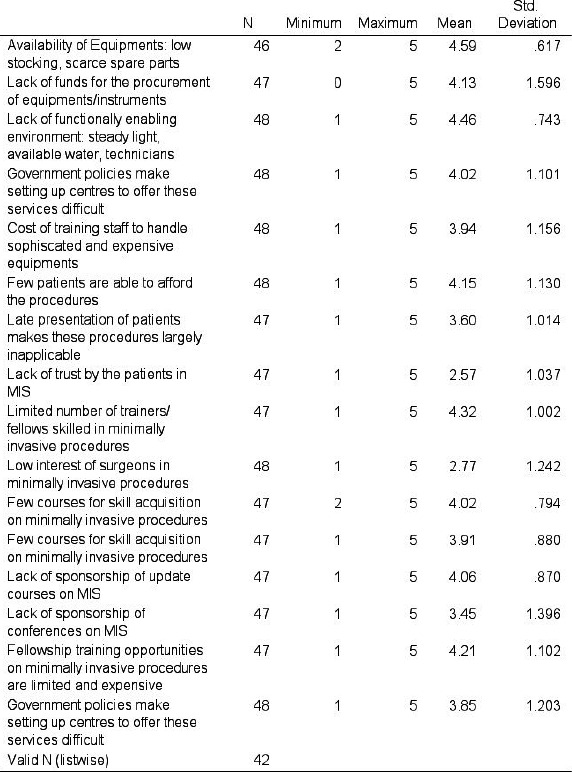
Challenges of minimally invasive orthopaedic surgery with mean and S.D values

**Table 1:** Socio-demographic factors

Factors	Frequency	Percentage
26-30	7	14.6
31-35	23	47.9
35-40	10	20.8
**Above 40**	8	16.7
		
**Gender**		
Male	4	8.3
Female	44	91.7
		
**Religion**		
Christianity	37	77.1
Islam	11	23.0
		
**Marital status**		
Married	28	58.3
Single	20	41.7
		
**Tribe**		
Hausa	7	14.6
Igbo	36	75.0
Yoruba	5	10.4

**Table 2:** showing participants range of opinions on possible challenges of minimally invasive Orthopedic surgery practices in Nigeria

S/N	Factors	Strongly agree n(%)	Agree n(%)	Not sure n(%)	Disagree n(%)	Strongly disagree n(%)
1	Availability of equipment: Low stocking, scarce material parts	29(60.4%)	18(37.5%)	0(0%)	1(2.1%)	0(0%)
2	Lack of funds for the procurement of equipment/instruments	35(72.9%)	5(10.4%)	1(2.1%)	2(4.2%)	5(10.4%)
3	Lack of functionally enabling environment: steady light, available water, technicians	26(54.2%)	20(41.7%)	1(2.1%)	0(0%)	1(2.1%)
4	Government policies make setting up centres to offer these services difficult	19(39.6%)	19(39.6%)	4(8.4%)	4(8.4%)	2(4.2%)
5	Cost of training staff to handle sophisticated and expensive equipment	16(33.3%)	24(50.0%)	0(0%)	5(10.4%)	3(6.3%)
6	Few patients are able to afford the procedure	24(50.0%)	15(31.3%)	3(6.3%)	4(8.3%)	2(4.2%)
7	Late presentation of patients make these procedures largely inapplicable	9(18.8%)	18(37.5%)	14(29.2%)	6(12.5%)	1(2.1%)
8	Lack of trust by the patients in MIS	1(2.1%)	10(20.8%)	10(20.8%)	21(43.8%)	6(12.5%)
9	Limited number of trainers/fellows skilled in minimally invasive procedures	26(54.2%)	16(33.3%)	3(6.3%)	1(2.1%)	2(2.4%)
10	Low interest of surgeons in minimally invasive surgery	4(8.3%)	14(29.2%)	3(6.3%)	21(43.8%)	6(12.5%)
11	Few courses for skill acquisition in minimally invasive surgery	24(60%)	26(54.2%)	8(33.5%)	4(8.4%)	1(2.1%)
12	Lack of sponsorship for update courses in MIS	13(27.1%)	29(60.4%)	1(2.1%)	4(8.4%)	1(2.1%)
13	Lack of sponsorship for conference in MIS	8(16.7%)	27(56.3%)	2(2.4%)	1(2.1%)	10(20.8%)
14	Fellowship training opportunities in Minimally invasive proceduresis limited and expensive	23(47.9%)	19(39.6%)	0(0%)	3(6.3%)	3(6.3%)
15	Government policies make setting up centres to offer these services difficult	17(35.4%)	18(37.5%)	5(10.4%)	5(10.4%)	3(6.3%)

## Data Availability

All forms of data and analysis are available on request to the corresponding author.

## Strength and limitations

It was a national survey, hence good representative with a good response rate, however further studies with larger response rate would be useful. Most forms of survey research bias was minimized. Internet challenges in a low middle income limiting some participants cannot be ignored and further studies can improve on this.

## References

[1] Hernández-Vaquero D, Fernández-Fairen M, Torres-Perez A, Santamariá A (2012). Minimally invasive surgery versus conventional surgery. A review of the scientific evidence. Rev Esp Cir Ortop Traumatol.

[2] Ijah RF, Ray-Offor E, Igwe PO, Ekeke ON, Okoro PE, Tamunomie N (2022). Minimally Invasive Surgery in Port Harcourt, Nigeria: Progress So Far. Cureus.

[3] Song Chengli (2010). History and Current Situation of Shape Memory Alloys Devices for Minimally Invasive Surgery. The Open Medical Devices Journal.

[4] Ijah RF, Okoro PE, Aaron FE, Tamunomie N, Omodu JO, Ocheli E (2021). Challenges of Minimally Invasive Surgery in a Southern Nigerian State: Issues for Discussion. Clinical casereports J.

[5] Mohiuddin K, Swanson SJ (2013). Maximizing the benefit of minimally invasive surgery. J Surg Oncol.

[6] Hughes-Hallett A, Mayer EK, Pratt PJ, Vale JA, Darzi AW (2015). Quantitative analysis of technological innovation in minimally invasive surgery. Br J Surg.

[7] Siddaiah-Subramanya M, Tiang KW, Nyandowe M (2017). A New Era of Minimally Invasive Surgery: Progress and Development of Major Technical Innovations in General Surgery Over the Last Decade. Surg J. (N Y).

[8] Costa DM, Lopes LKO, Vickery K, Watanabe E, Vasconcelos LSNOL, de Paula MC (2018). Reprocessing safety issues associated with complex-design orthopaedic loaned surgical instruments and implants. Injury.

[9] Treuting R (2000). Minimally invasive orthopedic surgery: arthroscopy. Ochsner J.

[10] Seyler TM, Johnson AJ, Marker DR, Mont MA, Bonutti PM (2011). Arthroscopic-assisted minimally invasive total knee arthroplasty. Arthroscopy.

